# Supplementary material for: Site-Directed Mutations at Phosphorylation Sites in Zea mays PHO1 Reveal Modulation of Enzymatic Activity by Phosphorylation at S566 in the L80 Region
Source: Plants (Basel). 2023 Sep 8;12(18):3205. doi: 10.3390/plants12183205 (PMC10536461; doi:10.3390/plants12183205)
Supplement: Supplementary file 1 [file plants-12-03205-s001.zip › plants-2536168-supplementary.pdf]

Supplementary material

Site-directed Mutations at Phosphorylation Sites in *Zea mays* PHO1 Reveal Modulation of Enzymatic Activity by Phosphorylation at S566 in the L80 Region

Noman Shoaib [1,2,3,†], Nishbah Mughal [1,†], Lun Liu [1,2], Ali Raza [3], Leiyang Shen[1,2], Guowu Yu [1,2,*]

***Correspondence:**
Guowu Yu (10024@sicau.edu.cn)

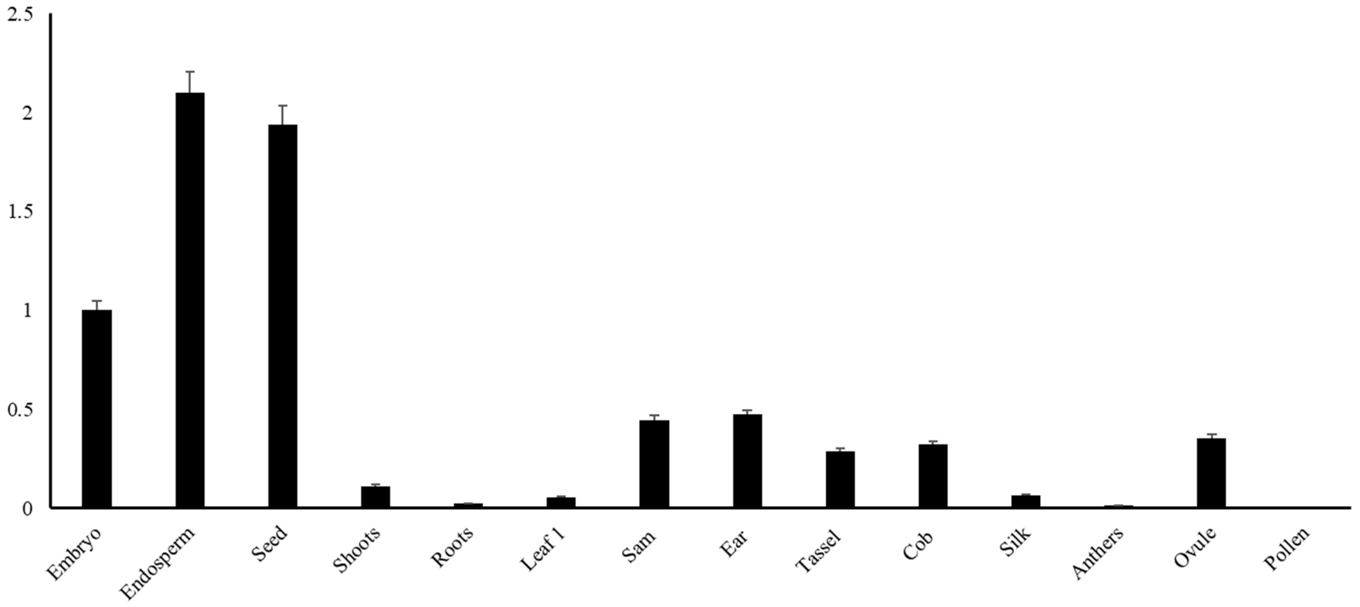

Figure S1: Tissue-specific expression levels of the PHO1 of *Zea mays*. The relative expression pattern is shown and the transcript level in the embryo is used as control. The leaf1 is the first leaf harvested from the mature plant when it is flowering. The 15 DAP seeds and dissected endosperm were used to analyze the transcript level.

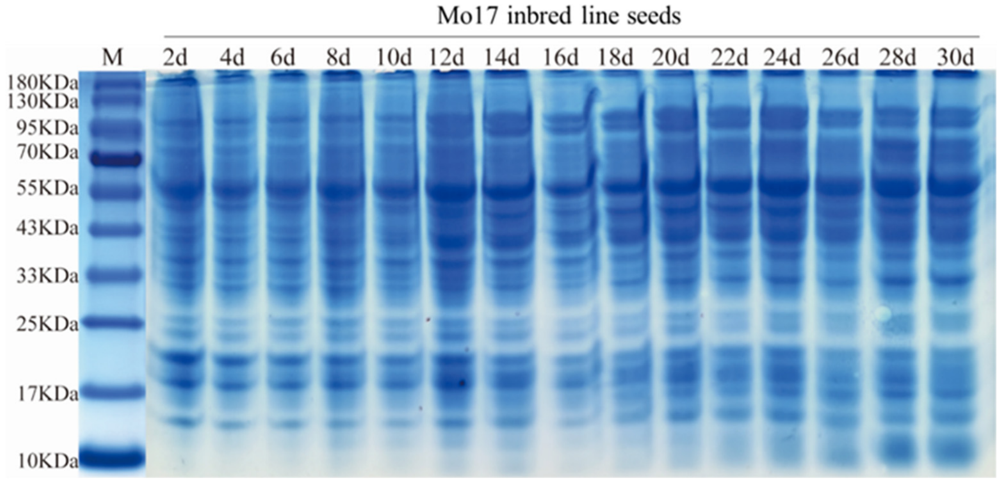

Figure S2. SDS-PAGE of overall protein distribution pattern. Amount of protein loaded was 30μg.

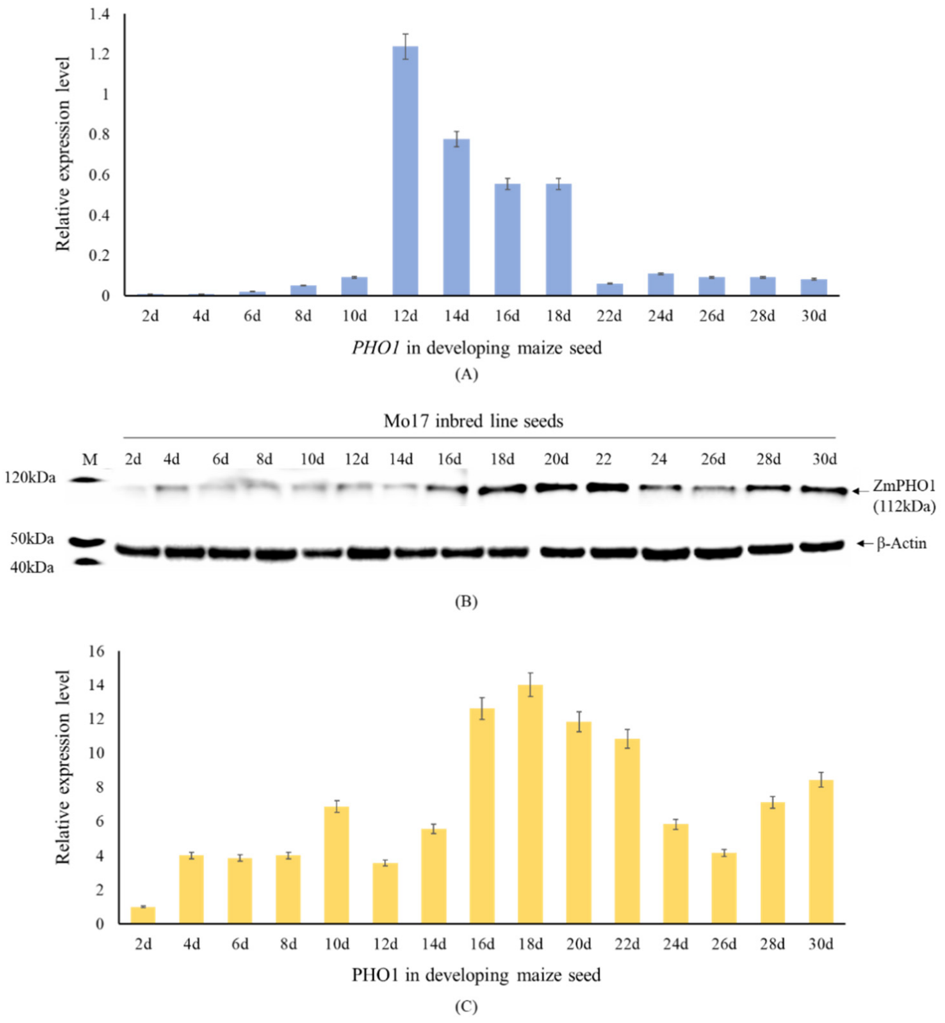

Figure S3. The expression level of *Zea mays* PHO1 (ZmPHO1) in different developmental stages of seed. (A) Relative expression levels of the ZmPHO1 transcripts in different developmental stages of the seeds. The transcript level in 2 DAP (days after pollination) seed was used as a control. (B) WB detection of ZmPHO1 protein expression pattern by using the antibody specific for ZmPHO1. The dilution ratio of the ZmPHO1 antibody was 1:1000 and the dilution ratio of β-Actin was 1:10000. The amount of protein loaded was 30μg. (C) Relative expression of ZmPHO1 protein in different developmental stages calculated through quantification of WB bands. ImageJ software with default settings (accessed on 5 January, 2022) was used to analyze the WB bands.

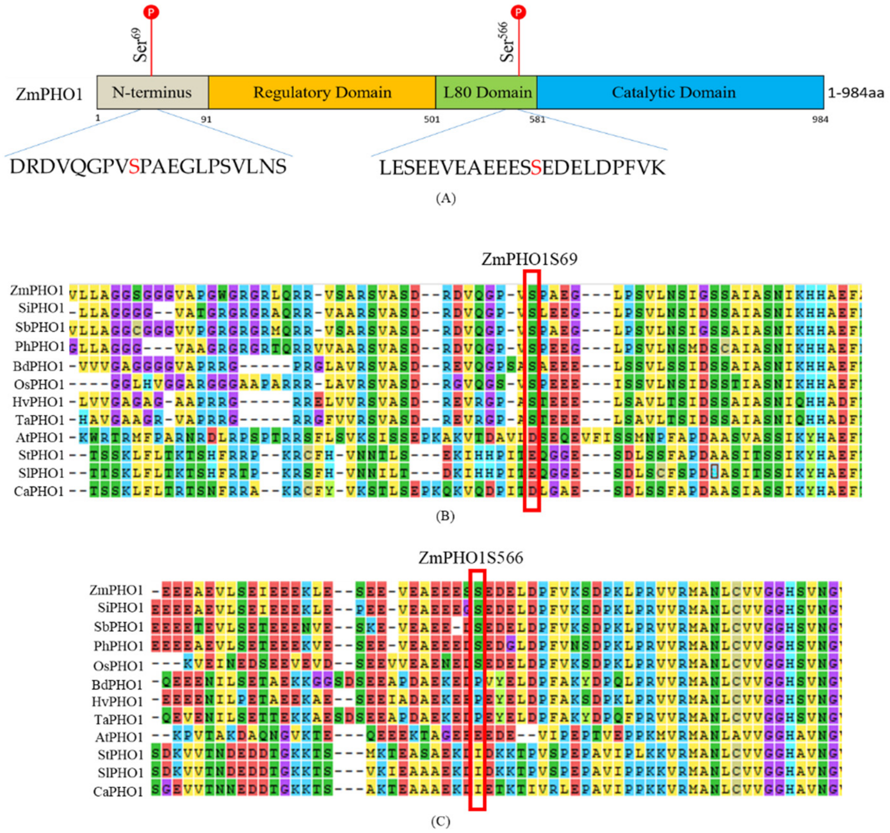

Figure S4. Representation and conservation of identified modifiable (phosphorylation) serine residues of PHO1. (A) Symbolic representation of phosphorylation sites on the primary structure of *Zea mays* PHO1 (ZmPHO1). Red letters are representing modifiable residue identified via iTRAQTM. (B) Conservation of Serine 69 (from *Zea mays*) phosphorylation site. (C) Conservation of Serine 566 (from *Zea mays*) phosphorylation site. Symbols include: Zm, *Zea mays* (accession id: NP_001296783.1); Sb, *Sorghum bicolor* (accession id: XP_021306483.1); Si, *Setaria italica* (accession id: XP_004981704.1); Ph, *Panicum hallii* (accession id: XP_025795811.1); Bd, *Brachypodium distachyon* (accession id: XP_003559211.1); Ta, *Triticum aestivum* (accession id: ACC59201.1); Hv, *Hordeum vulgare* (accession id: KAE8783983.1); Os, *Oryza sativa* (accession id: XP_015631420.1); At, *Arabidopsis thaliana* (accession id: Q9LIB2.1); Sl, *Solanum lycopersicum* (accession id: NP_001362574.1); St, *Solanum tuberosum* (accession id: NP_001275215.1); and Ca, *Capsicum annuum* (accession id: XP_016569840.1).

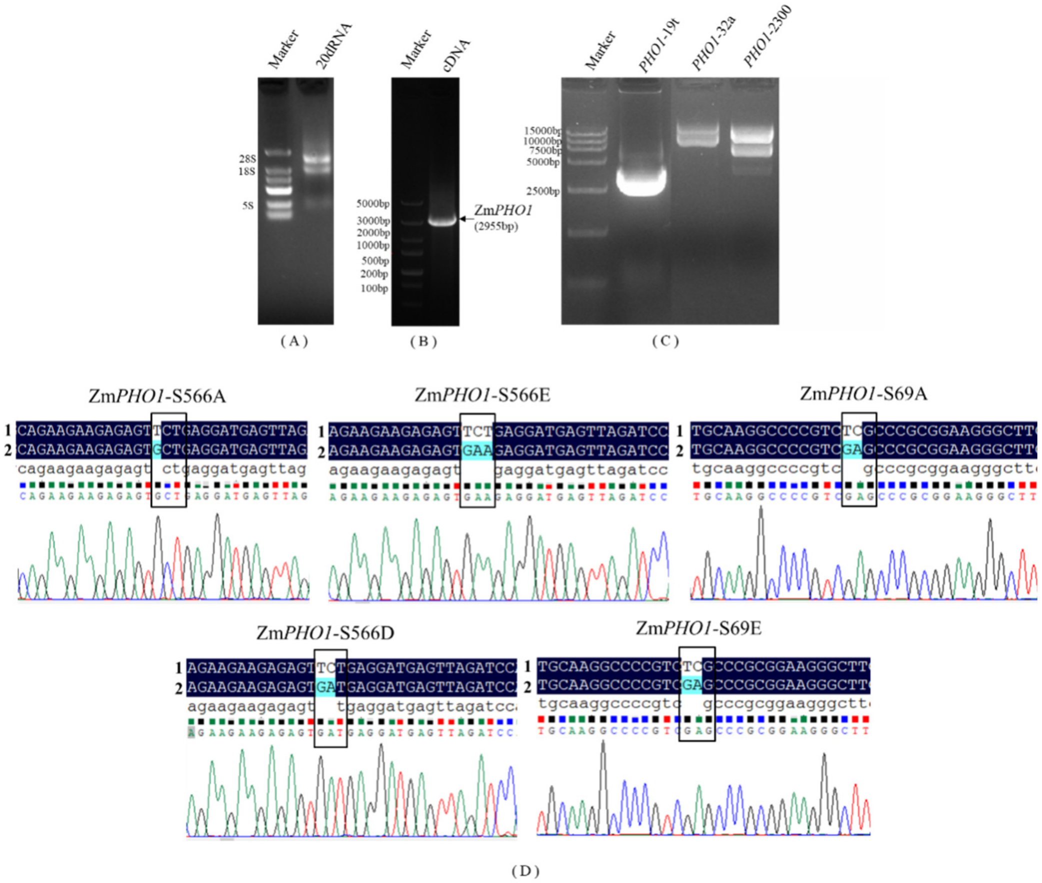

Figure S5. Amplification, cloning, and sequencing of Zm*PHO1* with various vectors. (A) Extracted RNA with 28S, 18S, and 5S bands. (B) Full-length amplified *PHO1*. (C) Cloned plasmids of *PHO1*. (D) Sequence results of *PHO1* mutation sites (connected with pMD19-T vector). 1: PHO1 sequences from the NCBI database. 2: mutated sequence results. Zm*PHO1*: PHO1 gene from *Zea mays*. S566A: serine coding base pairs (bp) at 566 amino acid (aa) position replaced with alanine coding bp. S566E: serine coding bp at 566 aa position replaced with glutamic acid coding bp. S566D: serine coding bp at 566 aa position replaced with aspartic acid coding bp. S69E: serine coding bp at 69 aa position replaced with glutamic acid coding bp. S69A: serine coding bp at 566 aa position replaced with alanine coding bp.

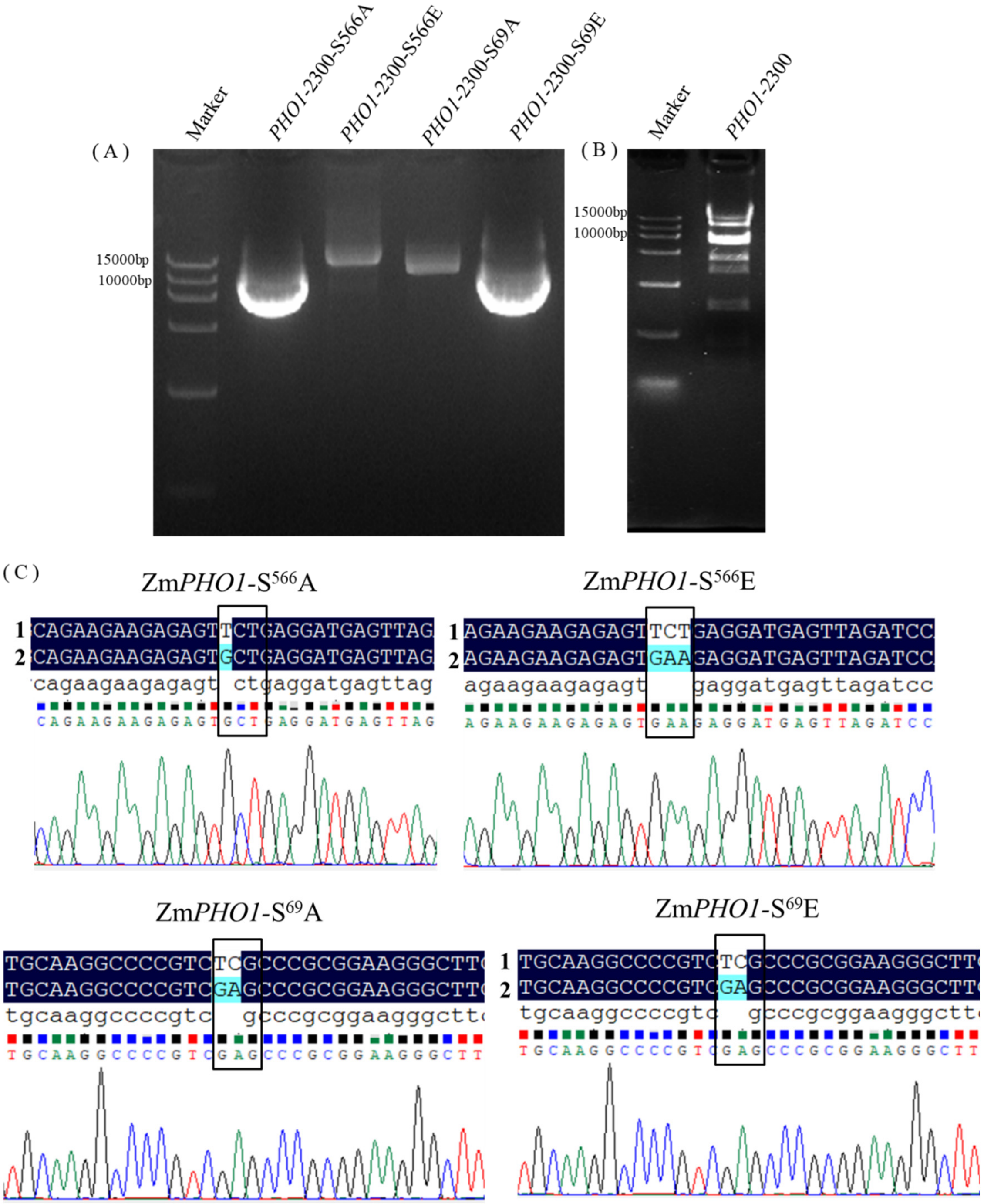

Figure S6. Cloning of Zm*PHO1* to pCAMBIA-2300-eGFP and sequence verification. (A) Amplified successful clones after plasmid extraction. (B) *PHO1*-2300 double digested with *Kpn* I and *Xba* I. (C) Sequence results of PHO1 mutation sites. 1: PHO1 sequences from NCBI database. 2: mutated sequence results. ZmPHO1: PHO1 gene from *Zea mays*. S566A: serine coding base pairs (bp) at 566 amino acid (aa) position replaced with alanine coding bp. S566E: serine coding bp at 566 aa position replaced with glutamic acid coding bp. S69E: serine coding bp at 69 aa position replaced with glutamic acid coding bp. S69A: serine coding bp at 566 aa position replaced with alanine coding bp.

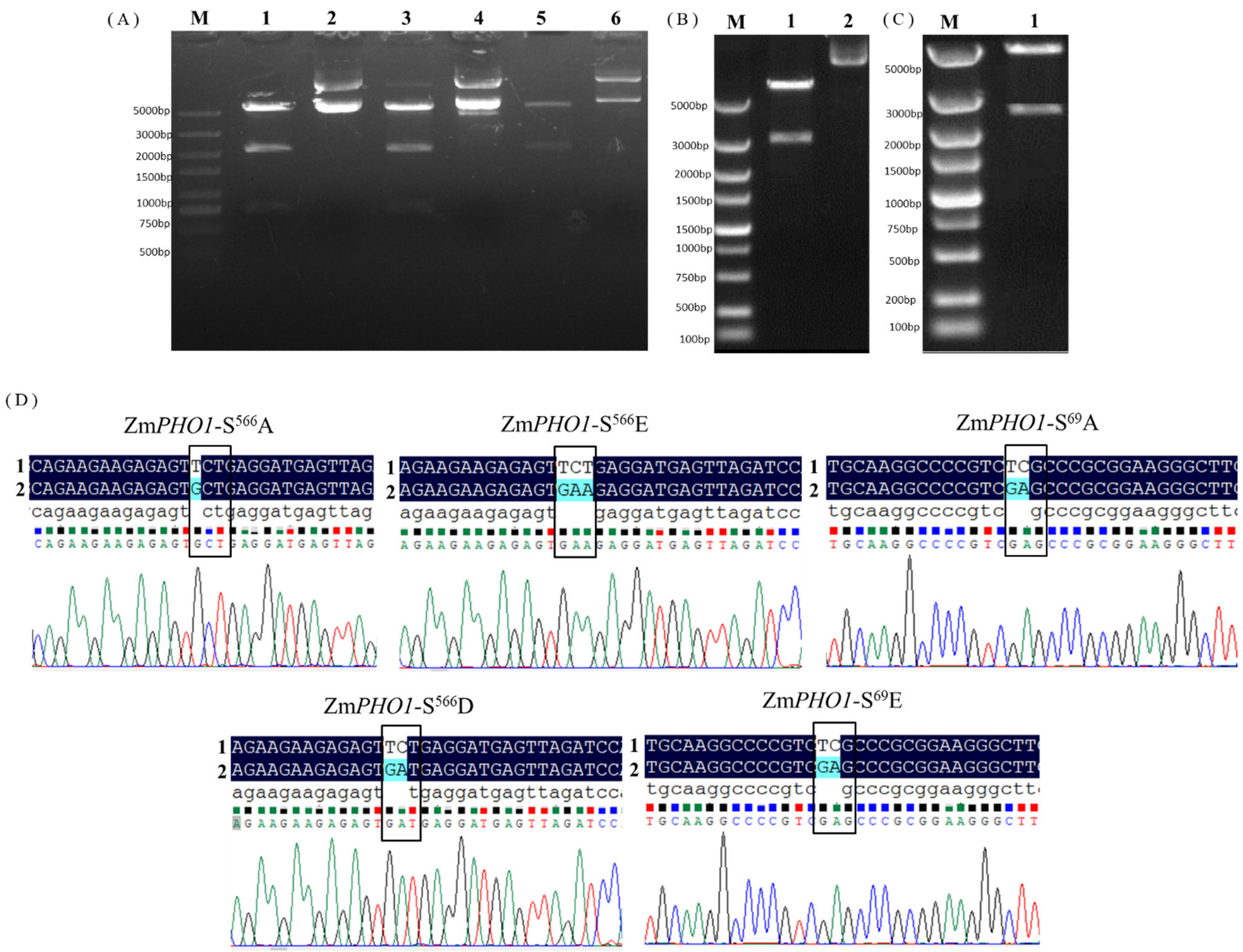

Figure S7. Cloning of Zm*PHO1* to pET32a vector, identification and sequence verification. (A) Amplified successful clones after plasmid extraction. M: marker, 1: Zm*PHO1*-32a-S566A double digested with *Eco*R I and *Eco*R V, 2: Zm*PHO1*-32a-S566A plasmid, 3: Zm*PHO1*-32a-S566D double digested with *Eco*R I and *Eco*R V, 4: Zm*PHO1*-32a-S566D plasmid, 5: Zm*PHO1*-32a-S566E double digested with *Eco*RI and *Eco*R V, 6: Zm*PHO1*-32a-S566E plasmid. (B) Amplified successful clones after plasmid extraction. M: marker, 1: Zm*PHO1*-32a-S69E double digested with EcoR1 and EcoRV, 2: Zm*PHO1*-32a-S69E plasmid. (C) Amplified successful clones after plasmid extraction. M: marker, 1: *PHO1*-AD-S69A double digested with *Eco*R I and *Eco*R V. (D) Sequence results of PHO1 mutation sites. 1: PHO1 sequences from NCBI database. 2: mutated sequence results. ZmPHO1: PHO1 gene from *Zea mays*. S566A: serine coding base pairs (bp) at 566 amino acid (aa) position replaced with alanine coding bp. S566E: serine coding bp at 566 aa position replaced with glutamic acid coding bp. S69E: serine coding bp at 69 aa position replaced with glutamic acid coding bp. S69A: serine coding bp at 566 aa position replaced with alanine coding bp.

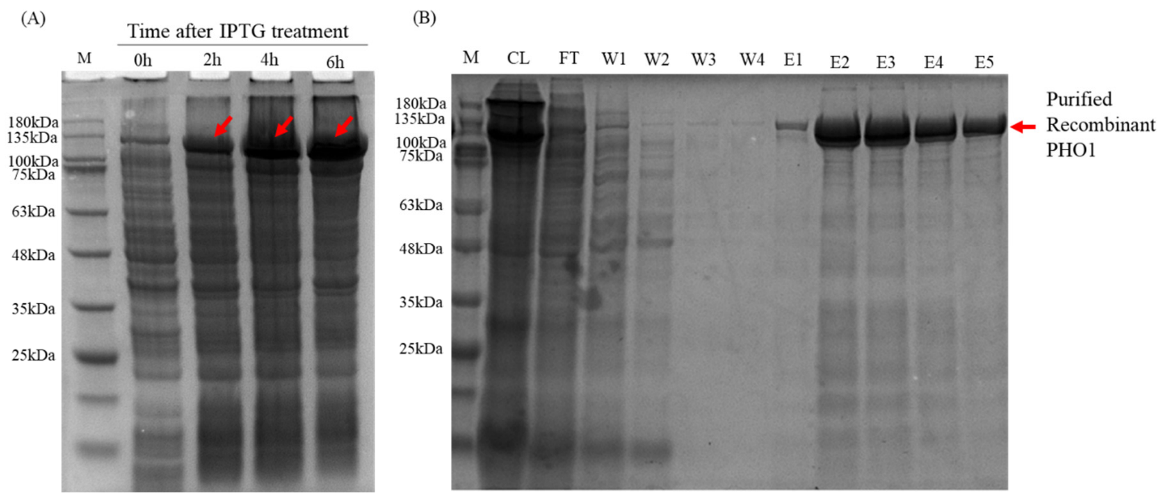

Figure S8. Expression and purification of recombinant PHO1. Symbols include: M; Marker, CL; Expressed protein overall, FT; Flow through, W1-W3; Washing buffer, E1-E5; Elutes. Red arrows are indicating expressed recombinant PHO1 bands. The amount of protein loaded was 30μg.

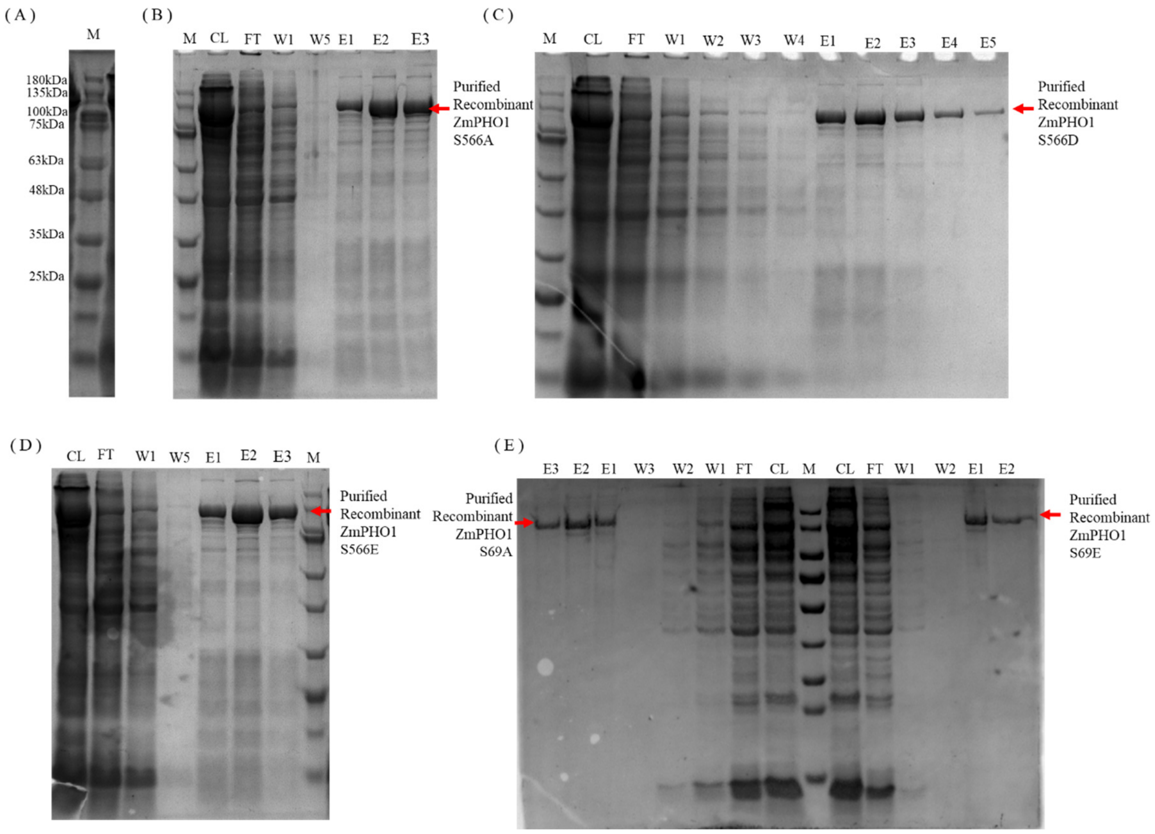

Figure S9. Expression and purification of recombinant PHO1. Symbols include: M; Marker, CL; Expressed protein overall, FT; Flow through, W1-W3; Washing buffer, E1-E5; Elutes. Red arrows are indicating expressed recombinant PHO1 bands. The amount of protein loaded was 30µg. (A) Marker. (B) Expression and purification of recombinant PHO1-S566A. (C) Expression and purification of recombinant PHO1-S566D. (D) Expression and purification of recombinant PHO1-S566E. (E) Expression and purification of recombinant PHO1-S69A and S69E.

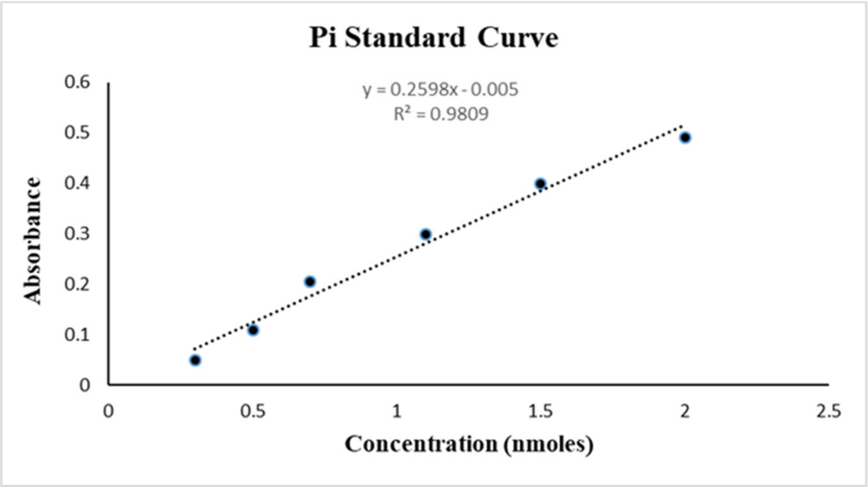

Figure S10. Inorganic phosphate Pi standard curve for the estimation of mutated and non-mutated recombinant PHO1 activity.

Table S1 Bioinformatics prediction of serine and threonine phosphorylation sites in the N-terminus and L80-Domain of ZmPHO1.

# Peptide	#	x	Context	Score	Kinase	Answer
# N-terminus	3	T	--MATTTSP	0.637	PKC	YES
# N-terminus	3	T	--MATTTSP	0.444	GSK3	.
# N-terminus	3	T	--MATTTSP	0.440	CaM-II	.
# N-terminus	3	T	--MATTTSP	0.401	p38MAPK	.

# N-terminus	3	T	--MATTTSP	0.369	cdc2	.
# N-terminus	4	T	-MATTTSPP	0.841	unsp	YES
# N-terminus	4	T	-MATTTSPP	0.527	cdc2	YES
# N-terminus	4	T	-MATTTSPP	0.459	GSK3	.
# N-terminus	4	T	-MATTTSPP	0.452	CaM-II	.
# N-terminus	4	T	-MATTTSPP	0.379	PKC	.
# N-terminus	5	T	MATTTSPPL	0.659	unsp	YES
# N-terminus	5	T	MATTTSPPL	0.426	GSK3	.
# N-terminus	5	T	MATTTSPPL	0.424	CaM-II	.
# N-terminus	5	T	MATTTSPPL	0.384	cdc2	.
# N-terminus	5	T	MATTTSPPL	0.379	PKC	.
# N-terminus	6	S	ATTTSPPLQ	0.580	cdk5	YES
# N-terminus	6	S	ATTTSPPLQ	0.511	cdc2	YES
# N-terminus	6	S	ATTTSPPLQ	0.503	p38MAPK	YES
# N-terminus	6	S	ATTTSPPLQ	0.493	GSK3	.
# N-terminus	6	S	ATTTSPPLQ	0.441	CaM-II	.
# N-terminus	6	S	ATTTSPPLQ	0.384	PKA	.
# N-terminus	6	S	ATTTSPPLQ	0.375	CKI	.
# N-terminus	13	S	LQLASASRP	0.606	cdc2	YES
# N-terminus	13	S	LQLASASRP	0.459	CaM-II	.
# N-terminus	13	S	LQLASASRP	0.457	GSK3	.
# N-terminus	15	S	LASASRPHA	0.547	cdc2	YES
# N-terminus	15	S	LASASRPHA	0.445	GSK3	.
# N-terminus	15	S	LASASRPHA	0.429	CaM-II	.
# N-terminus	15	S	LASASRPHA	0.369	DNAPK	.
# N-terminus	22	S	HASASGGGG	0.636	unsp	YES
# N-terminus	22	S	HASASGGGG	0.541	CKI	YES
# N-terminus	22	S	HASASGGGG	0.471	cdc2	.
# N-terminus	22	S	HASASGGGG	0.445	GSK3	.
# N-terminus	22	S	HASASGGGG	0.436	CaM-II	.
# N-terminus	22	S	HASASGGGG	0.401	PKC	.
# N-terminus	22	S	HASASGGGG	0.354	DNAPK	.
# N-terminus	36	S	LAGGSGGGV	0.535	CKI	YES
# N-terminus	36	S	LAGGSGGGV	0.476	CaM-II	.
# N-terminus	36	S	LAGGSGGGV	0.351	DNAPK	.
# N-terminus	54	S	QRRVSARSV	0.995	unsp	YES
# N-terminus	54	S	QRRVSARSV	0.653	PKA	YES
# N-terminus	54	S	QRRVSARSV	0.615	RSK	YES
# N-terminus	54	S	QRRVSARSV	0.504	PKG	YES
# N-terminus	54	S	QRRVSARSV	0.492	CaM-II	.
# N-terminus	54	S	QRRVSARSV	0.463	cdc2	.
# N-terminus	54	S	QRRVSARSV	0.437	GSK3	.
# N-terminus	57	S	VSARSVASD	0.987	unsp	YES
# N-terminus	57	S	VSARSVASD	0.532	PKC	YES
# N-terminus	57	S	VSARSVASD	0.339	CKII	.
# N-terminus	60	S	RSVASDRDV	0.989	unsp	YES
# N-terminus	60	S	RSVASDRDV	0.711	PKC	YES
# N-terminus	60	S	RSVASDRDV	0.430	GSK3	.
# N-terminus	60	S	RSVASDRDV	0.423	CaM-II	.
**# N-terminus**	**69**	**S**	**QGPVSPAEG**	**0.993**	**unsp**	**YES**
**# N-terminus**	**69**	**S**	**QGPVSPAEG**	**0.528**	**p38MAPK**	**YES**
# N-terminus	69	S	QGPVSPAEG	0.493	GSK3	.
# N-terminus	69	S	QGPVSPAEG	0.486	CKII	.
# N-terminus	69	S	QGPVSPAEG	0.478	cdc2	.
# N-terminus	69	S	QGPVSPAEG	0.438	CaM-II	.
# N-terminus	69	S	QGPVSPAEG	0.416	cdk5	.
# N-terminus	69	S	QGPVSPAEG	0.363	CKI	.
# N-terminus	76	S	EGLPSVLNS	0.343	RSK	.
# N-terminus	84	S	SIGSSAIAS	0.697	PKC	YES
# N-terminus	84	S	SIGSSAIAS	0.468	GSK3	.
# N-terminus	84	S	SIGSSAIAS	0.441	CaM-II	.
# N-terminus	84	S	SIGSSAIAS	0.433	cdc2	.
# N-terminus	88	S	SAIASNIKH	0.856	PKC	YES
# N-terminus	88	S	SAIASNIKH	0.463	cdc2	.
# N-terminus	88	S	SAIASNIKH	0.452	GSK3	.
# N-terminus	88	S	SAIASNIKH	0.446	CaM-II	.
# N-terminus	88	S	SAIASNIKH	0.359	DNAPK	.

#	N-terminus	88 S	SAIASNIKH	0.356	CKI	.
#	L80-Domain	502 S	PASISQLFV	0.509	ATM	YES
#	L80-Domain	502 S	PASISQLFV	0.507	cdc2	YES
#	L80-Domain	502 S	PASISQLFV	0.468	CaM-II	.
#	L80-Domain	502 S	PASISQLFV	0.444	DNAPK	.
#	L80-Domain	502 S	PASISQLFV	0.423	GSK3	.
#	L80-Domain	502 S	PASISQLFV	0.394	CKI	.
#	L80-Domain	502 S	PASISQLFV	0.304	CKII	.
#	L80-Domain	514 S	DKKESPAKS	0.994	unsp	YES
#	L80-Domain	514 S	DKKESPAKS	0.536	p38MAPK	YES
#	L80-Domain	514 S	DKKESPAKS	0.505	GSK3	YES
#	L80-Domain	514 S	DKKESPAKS	0.481	RSK	.
#	L80-Domain	514 S	DKKESPAKS	0.430	CaM-II	.
#	L80-Domain	514 S	DKKESPAKS	0.428	cdk5	.
#	L80-Domain	518 S	SPAKSKQKL	0.584	unsp	YES
#	L80-Domain	518 S	SPAKSKQKL	0.473	GSK3	.
#	L80-Domain	518 S	SPAKSKQKL	0.455	CaM-II	.
#	L80-Domain	518 S	SPAKSKQKL	0.383	cdc2	.
#	L80-Domain	518 S	SPAKSKQKL	0.374	RSK	.
#	L80-Domain	518 S	SPAKSKQKL	0.344	DNAPK	.
#	L80-Domain	518 S	SPAKSKQKL	0.330	cdk5	.
#	L80-Domain	526 S	LLVKSLETI	0.725	unsp	YES
#	L80-Domain	526 S	LLVKSLETI	0.459	GSK3	.
#	L80-Domain	526 S	LLVKSLETI	0.444	CaM-II	.
#	L80-Domain	526 S	LLVKSLETI	0.394	DNAPK	.
#	L80-Domain	529 T	KSLETIVDV	0.637	unsp	YES
#	L80-Domain	529 T	KSLETIVDV	0.484	CKII	.
#	L80-Domain	529 T	KSLETIVDV	0.466	CaM-II	.
#	L80-Domain	529 T	KSLETIVDV	0.440	CKI	.
#	L80-Domain	537 T	VEEKTELEE	0.854	unsp	YES
#	L80-Domain	537 T	VEEKTELEE	0.747	CKII	YES
#	L80-Domain	537 T	VEEKTELEE	0.434	GSK3	.
#	L80-Domain	537 T	VEEKTELEE	0.429	cdc2	.
#	L80-Domain	537 T	VEEKTELEE	0.427	CaM-II	.
#	L80-Domain	537 T	VEEKTELEE	0.382	CKI	.
#	L80-Domain	537 T	VEEKTELEE	0.337	DNAPK	.
#	L80-Domain	547 S	AEVLSEIEE	0.985	unsp	YES
#	L80-Domain	547 S	AEVLSEIEE	0.757	CKII	YES
#	L80-Domain	547 S	AEVLSEIEE	0.568	CKI	YES
#	L80-Domain	547 S	AEVLSEIEE	0.481	cdc2	.
#	L80-Domain	547 S	AEVLSEIEE	0.442	GSK3	.
#	L80-Domain	547 S	AEVLSEIEE	0.419	CaM-II	.
#	L80-Domain	556 S	EKLESEEVE	0.973	unsp	YES
#	L80-Domain	556 S	EKLESEEVE	0.691	CKII	YES
#	L80-Domain	556 S	EKLESEEVE	0.455	CKI	.
#	L80-Domain	556 S	EKLESEEVE	0.444	CaM-II	.
#	L80-Domain	556 S	EKLESEEVE	0.307	ATM	.
#	L80-Domain	565 S	AEEESSEDE	0.996	unsp	YES
#	L80-Domain	565 S	AEEESSEDE	0.693	CKII	YES
#	L80-Domain	565 S	AEEESSEDE	0.592	CKI	YES
#	L80-Domain	565 S	AEEESSEDE	0.463	GSK3	.
#	L80-Domain	565 S	AEEESSEDE	0.435	CaM-II	.
#	L80-Domain	565 S	AEEESSEDE	0.426	cdc2	.
**#**	**L80-Domain**	**566 S**	**EEESSEDEL**	**0.993**	**unsp**	**YES**
**#**	**L80-Domain**	**566 S**	**EEESSEDEL**	**0.747**	**CKII**	**YES**
**#**	**L80-Domain**	**566 S**	**EEESSEDEL**	**0.517**	**CKI**	**YES**
#	L80-Domain	566 S	EEESSEDEL	0.405	cdc2	.
